# SCN1Bβ mutations that affect their association with Kv4.3 underlie early repolarization syndrome

**DOI:** 10.1111/jcmm.13839

**Published:** 2018-08-30

**Authors:** Hao Yao, Jun Fan, Yun‐Jiu Cheng, Xu‐Miao Chen, Cheng‐Cheng Ji, Li‐Juan Liu, Zi‐Heng Zheng, Su‐Hua Wu

**Affiliations:** ^1^ Department of Cardiology the First Affiliated Hospital Sun Yat‐Sen University and Key Laboratory on Assisted Circulation NHC Guangzhou China

**Keywords:** early repolarization syndrome, SCN1Bβ, transient outward potassium current

## Abstract

**Background:**

Abnormal cardiac ion channels current, including transient outward potassium current (I_to_), is associated with early repolarization syndrome (ERS). Previous studies showed that mutations in SCN1Bβ both to increase the I_to_ current and to decrease the sodium current. Yet its role in ERS remains unknown.

**Objective:**

To determine the role of mutations in the SCN1Bβ subunits in ERS.

**Methods:**

We screened for mutations in the SCN1B genes from four families with ERS. Wild‐type and mutant SCN1Bβ genes were co‐expressed with wild‐type KCND3 in human embryonic kidney cells (HEK293). Whole‐cell patch‐clamp technique and co‐immunoprecipitation were used to study the electrophysiological properties and explore the underlying mechanisms.

**Results:**

S248R and R250T mutations in SCN1Bβ were detected in 4 families’ probands. Neither S248R nor R250T mutation had significant influence on the sodium channel current density (I_N__a_) when co‐expressed with SCN5A/WT. Co‐expression of KCND3/WT and SCN1Bβ/S248R or SCN1Bβ/R250T increased the transient outward potassium current I_to_ by 27.44% and 199.89%, respectively (*P *<* *0.05 and *P *<* *0.01, respectively) when compared with SCN1Bβ/WT. Electrophysiological properties showed that S248R and R250T mutations decreased the steady‐state inactivation and recovery from inactivation of I_to_ channel. Co‐immunoprecipitation study demonstrated an increased association between SCN1Bβ mutations and Kv4.3 compared with SCN1Bβ/WT (*P *<* *0.05 and *P *<* *0.01, respectively).

**Conclusion:**

The S248R and R250T mutations of SCN1Bβ gene caused gain‐of‐function of I_to_ by associated with Kv4.3, which maybe underlie the ERS phenotype of the probands.

## 1 INTRODUCTION

The early repolarization pattern (ERP) is characterized by an elevation of J‐wave >0.1 mV and sometimes involving an ST‐segment elevation in at least two contiguous leads. Recent studies have revealed that ERP is associated with a higher risk of malignant ventricular arrhythmia and sudden cardiac death (SCD).^1^, ^2^, ^3^, ^4^, ^5^ When a subject with ERP and malignant ventricular arrhythmias, that often known as early repolarization syndrome (ERS). Since the high prevalence of ERP in the general population (1.3%‐9.2%),^6^, ^7^, ^8^, ^9^ especially in younger physically active individuals^10^, ^11^ and male sex,^12^ it is significant to determine whose individuals with such common electrophysiological pattern are at risk of sudden cardiac death.

Some studies revealed that cardiac ion channel mutations play a major role in the pathogenesis of malignant ERPs. Mutations in the inward‐rectifier ATP‐dependent K+ channel current (I_KATP_),^13^, ^14^, ^15^ L‐type calcium current (I_CaL_)^16^, ^17^ and transient outward potassium current (I_to_)^18^, ^19^ can lead to ERPs. In inherited families, mutations in seven different genes (KCNJ8, CACNA1C, KCND3, KChip2, SCN5A, ABCC9, Ankyrin‐2) have been associated with ERPs.^13^, ^14^, ^15^, ^16^, ^17^, ^20^, ^21^, ^22^, ^23^

The transient outward potassium channel (I_to_) is a multi‐subunit protein complex comprised of pore‐forming α‐subunits and auxiliary β‐subunits.^24^ In humans, Kv4.3 encodes the α‐subunits of I_to._ 25 In human ventricular cells, I_to_ currents mediate the early phase of action potential repolarization,^18^ which can reduce the action potential duration by accelerating the early repolarization velocity and progressively suppressing the voltage of plateau phase.^19^ Increasing of I_to_ currents in region during initial ventricular repolarization can result in a J‐wave on the ECG.^26^ Therefore, disorders in I_to_ currents might be underlying mechanism of ERS.

SCN1B encodes the cardiac sodium channel β‐subunit.^27^ It is comprised of large extracellular immunoglobulin‐like domains, a single transmembrane‐spanning segment, and intracellular C‐terminal domains. SCN1B has two kinds of transcripts, SCN1B and SCN1Bβ, which encodes Navβ1 and Navβ1b, respectively.^27^ Functional analysis indicated that β‐subunit encodes by SCN1B involved in modulation of sodium channel gating and voltage dependence,^28^, ^29^ expression of sodium channel at the cell surface,^30^ and cell adhesion.^31^

Here, we reported a mutation in the SCN1Bβ gene, which encodes the regulatory β‐subunits of the transient outward potassium current (I_to_), identified in four families with ERS. We studied whether mutant in the SCN1Bβ was associated with ERS and explore the possible mechanisms. Electrophysiology modification by SCN1Bβ mutant was evaluated using patch‐clamp technology.

## 2 METHODS

### 2.1 Genetic analysis

Genomic DNA used for genetic analysis was isolated from peripheral blood samples. The protein coding sequences of the SCN1B genes were amplified by polymerase chain reaction (PCR) and directed sequenced. The DNA sequence was then compared with the reference sequence of NM_001037 (SCN1B isoform a), NM_199037 (SCN1B isoform b) and NM_001321605 (SCN1B isoform c).

### 2.2 Plasmid constructions and cell transfection

Human embryonic kidney cells (HEK293) were obtained from Type Culture Collection of Chinese Academic of Sciences. HEK293 cells were cultured in DMEM medium supplemented with 10% fetal bovine serum (FBS). cDNA encoding Nav1.5 (SCN5A, NM_198056) was amplified by PCR and cloned into mammalian expression vector pENTER. SCN1bβ (NM_199037) cDNA was subcloned into pIRES2‐EGFP. The p.S248R and p.R250T mutations were introduced by site‐directed mutagenesis using the QuikChange II kit (Stratagene, CA, USA). Kv4.3 (KCND3, NM_004980) cDNA was subcloned into pcDNA3.1. Nav1.5 and SCN1Bβ or Kv4.3 and SCN1Bβ plasmids were transiently co‐transfected (1:1 molar ratio) into HEK293 cells using Lipofectamine 2000 (Invitrogen Life Technologies Inc., CA, USA) according to the manufacturer's instructions.

### 2.3 Electrophysiology

Electrophysiological studies were performed 48 hours after transfection. Membrane currents were recorded by whole‐cell patch‐clamp technologies using a EPC‐10 amplifier (HEKA Instruments, Lambrecht, Germany) at room temperature (20‐25°C). The extracellular solution contained (in mmol/L): 140 NaCl, 5 KCl, 1 MgCl_2_, 1.8 CaCl_2_, 10 HEPES, 10 Glucose (PH 7.40 with NaOH). Pipette solution for I_Na_ contained (in mmol/L): 120 CsF, 20 CsCl, 2 EGTA, 5 HEPES, 5 Na_2_‐ATP (PH 7.20 with CsOH). For I_to_ currents, pipette solution contained (in mmol/L): 110 K‐aspartate, 20 KCl, 2 MgCl_2_, 10 HEPES, 5 EGTA, 5 Na_2_‐ATP (PH 7.20 with KOH). Pipettes were pulled from borosilicate glass capillaries using a micropipette puller (P‐97, Sutter Instruments, CA, USA). The tip resistances of patch pipettes ranged from 3‐5MΩ when filled with the pipette solution. Currents were filtered with a four pole Bessel filter at 5 kHz and digitized at 50 kHz. Series resistance was electronically compensated at around 80%.

I_Na_ currents were elicited by depolarizing pulses ranging from −90 mV to +40 mV in 10 mV increments with a holding potential (HP) at −120 mV. Peak currents were measured and I_Na_ densities (pA/pF) were obtained by dividing the peak I_Na_ by the cell capacitance obtained. The plots of voltage dependent steady state activation and inactivation were fitted by Boltzmann equation: *y* = 1/[1 + exp(*V−V*_1/2_)/*k*], where *V*_1/2_ is the voltage at which sodium current is half‐maximally activated, and *k* was the slope factor. To assess the time course of recovery from inactivation, a prepulse to 0 mV for 20 ms was followed by a recovery interpulse of variable duration (from 0.25 to 750 ms) to −120 mV and then a 25 ms test pulse to 0 mV to determine the fraction of recovered channels. To analyse the kinetics of recovery from inactivation, the time constants were obtained by fitting to a double‐exponential equation: *y* = A*[1‐exp(−*t*/τ*f*)]+(1‐*A*)*[1‐exp(−*t/*τs)].

I_to_ currents were elicited from a HP of −80 mV with depolarizing voltage pulses from −80 mV to +80 mV for 400 ms. Current density (pA/pF) was calculated from the ratio of current amplitude to cell capacitance. Peak currents were normalized to the maximum peak I_to_ amplitude. Normalized activation and inactivation curves were fit with a Boltzmann equation: *y* = 1/[1 + exp(*V−V*_1/2_)/*k*], where *V*_1/2_ is the voltage at which sodium current is half‐maximally activated, and *k* was the slope factor. Recovery from inactivation was assessed by a standard paired pulse protocol: a 500 ms test pulse to +50 mV (P1) was followed by a variable recovery interval at 380 mV, then by a second test pulse to +50 mV (P2). The plot of P2/P1 was then fit with two exponential to determine the time constants for recovery, using the equation: *y* = A*[1‐exp(−*t/*τf)]+(1*‐A*)*[1*‐*exp(−*t*/τs)].

### 2.4 Co‐immunoprecipitation and western blot

48 hours after transfection, HEK293 cells were scraped off from 100 mm dishes. The membrane proteins were extracted using a Membrane Protein Extraction Kit (Thermo Fisher Scientific, Waltham, MA) according to the manufacturer's instructions. Cell membrane lysates were then incubated with anti‐Kv4.3 antibodies (Sigma, USA) overnight at 4°C with rotation. Immune complexes were incubated with Protein G Dynabeads (Life technologies, USA) for 1 hour at room temperature with rotation and washed three time with PBS including 0.02% Tween‐20 to removed unbound material. The final pellet was boiled in 1 × SDS buffer and separated by 10% SDS‐PAGE. Western blot analysis was carried out using anti‐Kv4.3 antibodies (1:1000, Sigma, USA) and anti‐SCN1Bβ antibodies (1:500, Abcam, USA). The density of each band was quantified using Image J software.

### 2.5 Statistical analysis

Data were presented as Mean ± SEM. Statistical comparisons were analysed using two‐tailed Student's *t*‐test and ANOVA with Student‐Newman‐Keuls test. A *P* value less than 0.05 was considered statistically significant.

## 3 RESULTS

### 3.1 Clinical data and genetic analysis

**Figure 1:**
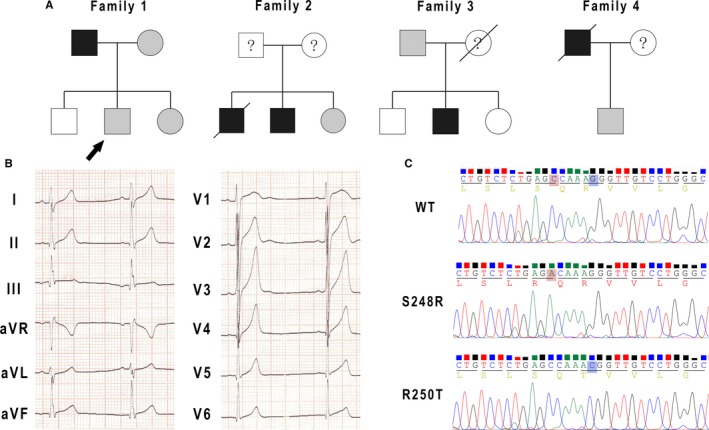
Genetic analyses and ECG in four families with early repolarization syndrome. A, Four family pedigrees with early repolarization syndrome (ERS). Black symbol: early repolarization pattern with ventricular fibrillation events. Grey symbol: early repolarization pattern without ventricular fibrillation events. B, Twelve‐lead ECG in a 14‐year‐old boy in Family 1 (arrow). C, DNA sequencing traces (chromatograms) for S248R and R250T variants identified in SCN1Bβ

Clinical data of the four families was showed in Table 1. Four family pedigrees with ERS were showed in Figure 1A. Figure 1B showed a 12‐lead ECG of a 14‐year‐old boy from Family 1 (arrow in Figure 1A). The ECG showed J‐point elevation in leads II, III and aVF. His father experienced sudden cardiac arrest at the age of 37 while chatting with others at afternoon. The emergency team recorded a ventricular fibrillation ECG from him and he was defibrillated immediately. There was no family history of SCD or syncope. The patient denied coronary artery disease, hypertension and diabetes mellitus. The echocardiogram was normal with no structural cardiac disease. No subsequent cardiac events have occurred over the next 8 years.

**Table 1:** Clinical characteristics of probands

ID	Sex	Age (y)	HR (bpm)	J‐wave location	J‐wave amplitude (mV)	ST elevation (mV)	R (mV)	QRS (ms)	QTc (ms)
1	M	25	66	*V*_1_‐*V*_4_	0.35	0.25	0.85	100	432
2	M	37	76	II, III, AVF, *V*_1_‐*V*_3_	0.1	0	1.5	80	360
3	M	32	72	I, aVL	0.1	0.1	0.9	60	394
4	M	71	75	II, III, AVF	0.1	0	0.8	70	391

Screening of SCN1Bβ in the four families revealed four mutations. Two were in UTR three of SCN1Bβ and two (c.C744A and c.G749C) were non‐synonymous mutation in exon three. Polymerase chain reaction (PCR) based sequencing analysis revealed a C‐to‐A replacement at nucleotide 744 and a G‐to‐C replacement at nucleotide 749 (Figure 1C), which result in a serine (S) to arginine (R) at residue 248 (S248R) and an arginine (R) to threonine (T) at residue 250 (R250T).

### 3.2 Electrophysiological characterization of SCN5A co‐expressed with SCN1Bβ/WT, SCN1Bβ/S248R and SCN1Bβ/R250T

**Figure 2:**
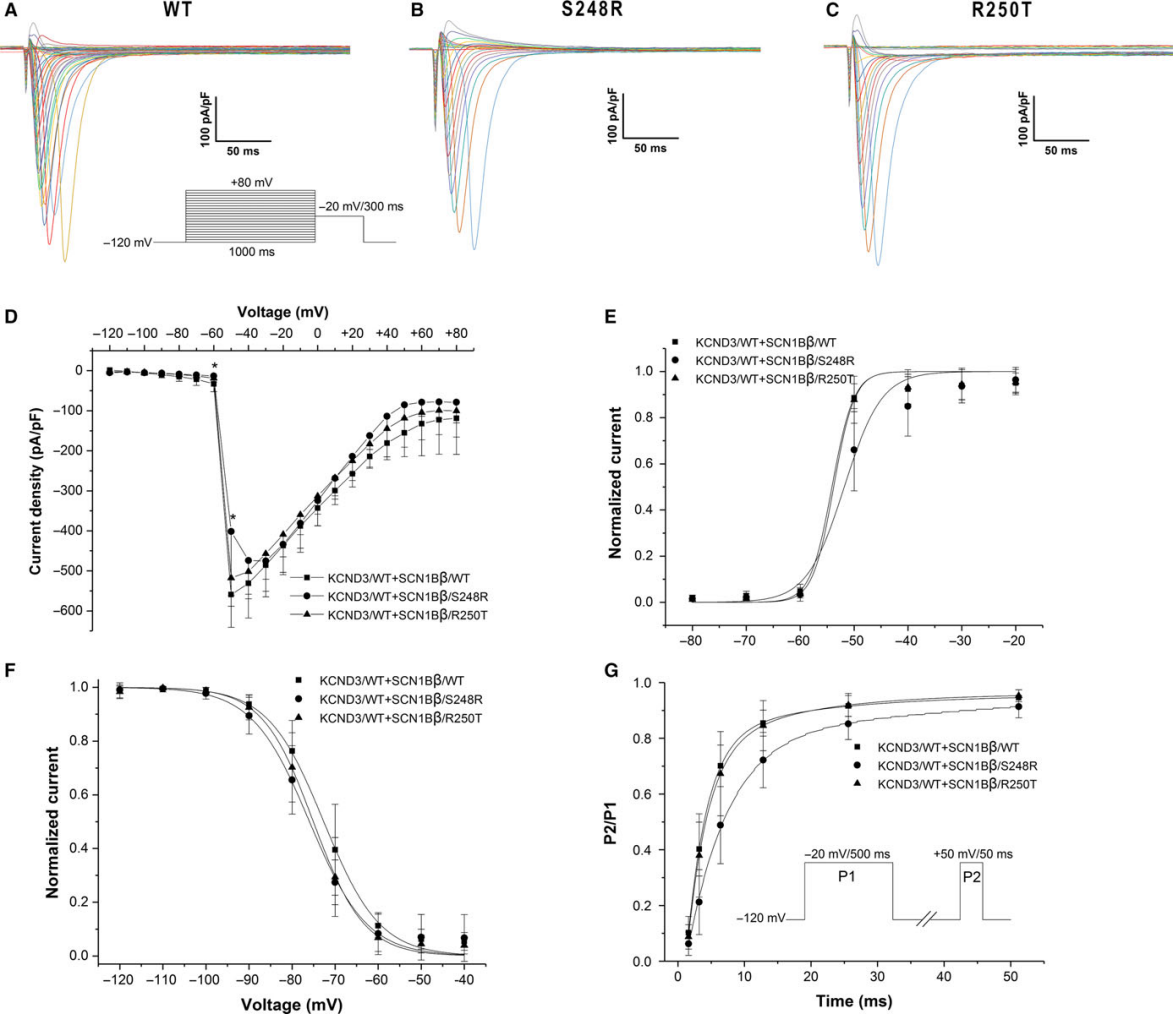
Sodium Currents recorded on HEK293 cells co‐expressed of SCN5A/WT and SCN1Bβ. A‐C, macroscopic currents recorded at *Vm* in the range −120 to +80 mV from a holding potential of −120 mV. Protocol used was shown in inset. D, Current‐voltage (I‐V) relationship of WT, S248R and R250T. (n = 9, 13 and 14, respectively, *P <* *0.05 vs WT). E, Voltage dependent steady‐state activation (*V*_1/2* *_= −54.10 ± 1.06 mV, n = 7 for WT; −51.81 ± 0.99 mV, n = 9 for S248R; −53.79 ± 1.02 mV, n = 6 for R250T). F: Voltage dependent steady‐state inactivation (*V*_1/2* *_= −72.62 ± 0.64 mV, n = 9 for WT; −75.94 ± 0.62 mV, n = 13 for S248R; −75.04 ± 0.37 mV, n = 12 for R250T). E and F plots were fitted by Boltzmann equation: *y* = 1/[1 + exp(*V−V*_1/2_)/*k*], *V*_1/2 _= voltage at which sodium current is half‐maximally activated, *k *= slope factor. G, Recovery from inactivation (n = 13, 14 and 14 for WT, S248R and R250T, respectively). Protocol used was shown in inset. The currents recorded at P2 were normalized to that at P1. Two‐exponential equation was used to fit the plot

In order to assess the effects of S248R and R250T mutation on Nav1.5 channel function, SCN5A/WT + SCN1Bβ/WT, SCN5A/WT + SCN1Bβ/S248R and SCN5A/WT + SCN1Bβ/R250T were expressed in HEK293 cells. Electrophysiological parameters of different channels were addressed by whole cell patch‐clamp technique. Figure 2A‐C showed macroscopic currents recorded at voltage in the range −120 to +80 mV from a holding potential of −120 mV. Figure 2D showed the voltage dependence of the averaged current density. S248R showed a decrease in the peak current density compared with WT (*P *<* *0.05). No significant difference in peak current density was observed in R250T compared with WT. Some minor differences were observed in steady‐state activation, inactivation and recovery from inactivation (Table 2). Both S248R and R250T showed a negative voltage shift of steady‐state inactivation compared with WT (Figure 2F. *V*_1/2 _= −72.62 ± 0.64 mV, n = 9 for WT; −75.94 ± 0.62 mV, n = 13 for S248R; −75.04 ± 0.37 mV, n = 12 for R250T; *P *<* *0.01 respectively.). S248R gave a minor positive voltage shift of steady‐state activation (*V*_1/2 _= −54.10 ± 1.06 mV, n = 7 for WT; −51.81 ± 0.99 mV, n = 9 for S248R; *P *<* *0.01) (Figure 2E). Recovery from inactivation was obtained using double pulse protocol and fitted with two‐exponential equation. τ_*f*_ was similar among WT and mutants, whereas S248R had a decreased in τ_*s*_ (18.02 ± 8.17, n = 13 for WT; 6.23 ± 0.86, n = 14 for S248R; *P *<* *0.01) (Figure 2G).

**Table 2:** Gating kinetics parameters of I_Na_ in HEK293 cells co‐expressed of SCN5A/WT and SCN1Bβ

Groups	Activation			Inactivation			Recovery		
	*V*_1/2_ (mV)	*k*	n	*V*_1/2_ (mV)	*k*	n	τ_*f*_ (ms)	τ_*s*_ (ms)	n
SCN5A/WT + SCN1Bβ/WT	−54.10 ± 1.06	2.01 ± 0.48	7	−72.62 ± 0.45	6.50 ± 0.40	9	2.98 ± 0.19	18.02 ± 8.17	13
SCN5A/WT + SCN1Bβ/S248R	−51.81 ± 0.99^a^	3.47 ± 1.00^a^	9	−75.94 ± 0.62^a^	6.67 ± 0.55	13	2.31 ± 1.99	6.23 ± 0.86^a^	14
SCN5A/WT + SCN1Bβ/R250T	−53.79 ± 1.02	1.93 ± 0.48	6	−75.04 ± 0.37^a^	5.91 ± 0.32^a^	12	3.04 ± 0.07	14.56 ± 1.45	14

Values are Mean ± SEM, *V*_1/2_: voltage of half‐maximally activated or inactivation, *k*: slope factor.

a *P *<* *0.01 vs WT.

### 3.3 Electrophysiological characterization of KCND3/WT co‐expressed with SCN1Bβ/WT, SCN1Bβ/S248R and SCN1Bβ/R250T

**Figure 3:**
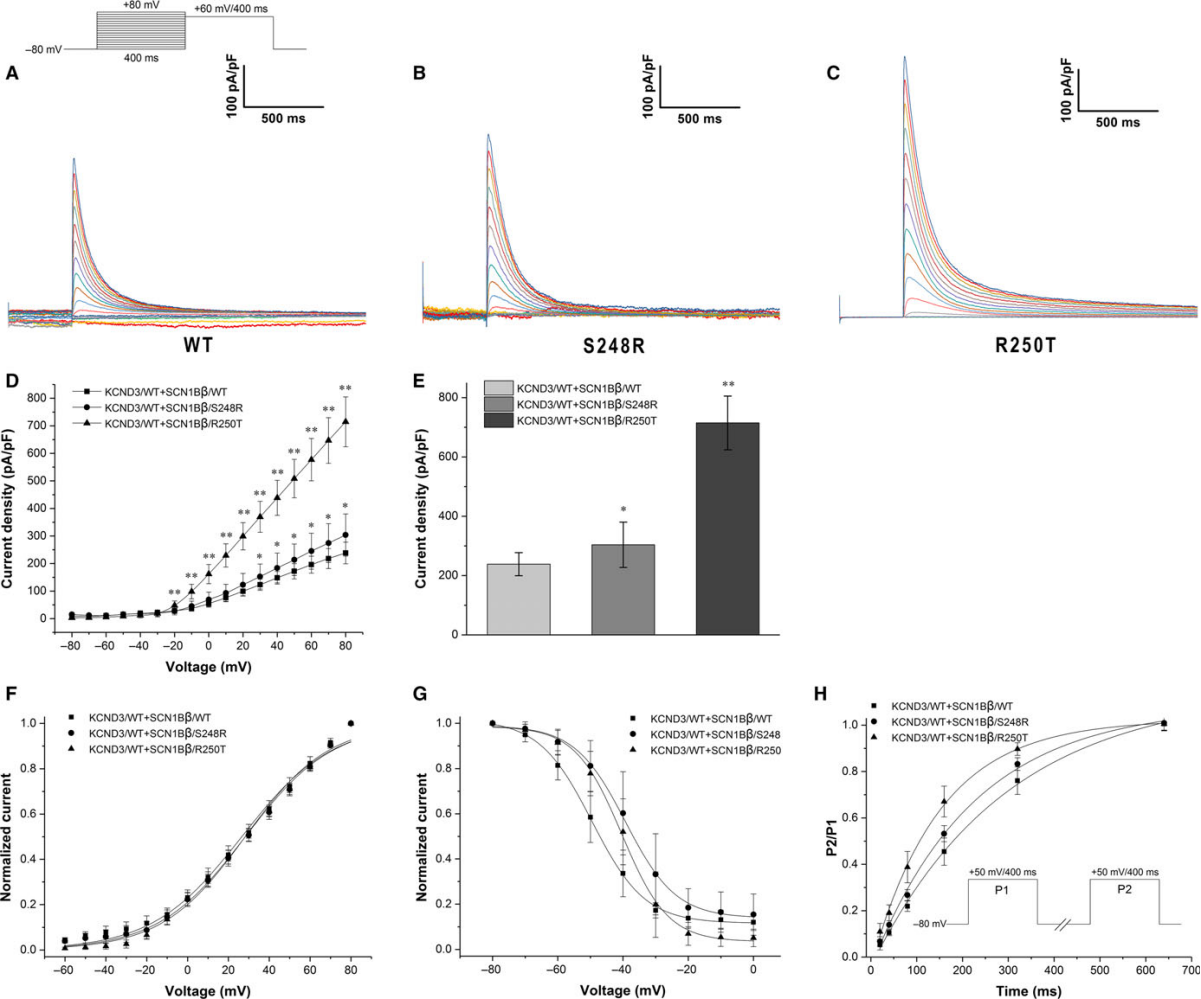
Transient outward potassium current recorded on HEK293 cells co‐expressed of KCND3/WT and SCN1Bβ. A‐C, macroscopic currents recorded at *Vm* in the range −80 to +80 mV from a holding potential of −80 mV. Protocol used was shown upside. D, Current‐voltage (I‐V) relationship of WT, S248R and R250T. (n = 9, 8 and 12, respectively. *P <* *0.05 vs WT. **P <* *0.01 vs WT). E, the mean peak I_to_ current densities recorded on repolarization to −80 mV from each group. *P <* *0.05 vs WT; **P <* *0.01 vs WT. F, Voltage dependent steady‐state activation (*V*_1/2 _= 26.58 ± 1.15 mV, n = 9 for WT; 28.29 ± 1.05 mV, n = 7 for S248R; 28.24 ± 1.04 mV, n = 12 for R250T). G, Voltage dependent steady‐state inactivation (*V*_1/2 _= −49.52 ± 0.41 mV, n = 9 for WT; −39.25 ± 0.61 mV, n = 8 for S248R; −40.39 ± 0.59 mV, n = 12 for R250T). F and G plots were fitted by Boltzmann equation: *y* = 1/[1 + exp(*V−V*_1/2_)/*k*], *V*_1/2_: voltage at which I_to_ is half‐maximally activated, *k *= slope factor. H, Recovery from inactivation (n = 8, 6 and 10 for WT, S248R and R250T respectively). Protocol used was shown in inset. The currents recorded at P2 were normalized to that at P1. Two‐exponential equation was used to fit the plot

To evaluate whether SCN1Bβ has an effect in modulating I_to_, we compared the electrophysiological properties of KCND3/WT co‐expressed with SCN1Bβ/WT, SCN1Bβ/S248R and SCN1Bβ/R250T in HEK293 cells. Figure 3A‐C displayed the representative current traces of WT and mutants. Compared with KCND3/WT + SCN1Bβ/WT, co‐expressed with mutants of SCN1Bβ showed an increase in peak current density (238.28 ± 39.12 pA/pF, n = 9 for WT; 303.67 ± 76.30 pA/pF, n = 8 for S248R; 714.57 ± 90.43 pA/pF, n = 13 for R250T. *P *<* *0.05 between WT and S248R. *P *<* *0.01 between WT and R250T). At ‐80 mV, co‐expressed of KCND3/WT with SCN1Bβ/S248R and SCN1Bβ/R250T result in an increase in I_to_ density by 27.44% and 199.89%, respectively (*P *<* *0.05 and *P *<* *0.01, respectively.) (Figure 3E). Steady‐state activation and inactivation were fitting with Boltzman equation. The *V*_1/2_ and slope factors were not statistically significant among WT and mutants (Table 3). Both S248R and R250T gave a positive shift in the steady‐state inactivation curve when compared to WT. (−49.52 ± 0.41 mV, n = 9 for WT; −39.25 ± 0.61 mV, n = 8 for S248R; −40.39 ± 0.59 mV, n = 12 for R250T; *P *<* *0.01 respectively). (Figure 3G). Figure 3H showed recovery curves of WT and mutants. Co‐expressed with SCN1Bβ/S248R gave slightly decrease in time constants when compared to SCN1Bβ/WT (295.11 ± 3.44, n = 8 for WT; 223.75 ± 2.04, n = 6 for S248R; *P *<* *0.01) (Table 3), whereas co‐expressed with SCN1Bβ/R250T result in a markedly reduce in time constants as compared with WT group (295.11 ± 3.44, n = 8 for WT; 150.18 ± 2.91, n = 10 for R250T, *P *<* *0.01).

**Table 3:** Gating kinetics parameters of I_to_ in HEK293 cells co‐expressed of KCND3/WT and SCN1Bβ

Groups	Activation			Inactivation			Recovery		
	*V*_1/2_ (mV)	*k*	n	*V*_1/2_ (mV)	*k*	n	τ_*f*_ (ms)	τ_*s*_ (ms)	n
KCND3/WT + SCN1Bβ/WT	26.58 ± 1.15	21.98 ± 1.07	9	−49.52 ± 0.41	8.12 ± 0.38	9	295.11 ± 3.44	295.11 ± 3.44	8
KCND3/WT + SCN1Bβ/S248R	28.29 ± 1.05	21.39 ± 0.98	7	−39.25 ± 0.61^a^	7.76 ± 0.57	8	223.75 ± 2.04^a^	223.75 ± 2.04^a^	6
KCND3/WT + SCN1Bβ/R250T	28.24 ± 1.04	20.41 ± 0.95	12	−40.39 ± 0.59^a^	7.10 ± 0.54^a^	12	150.18 ± 2.91^a^	150.18 ± 2.91^a^	10

Values are Mean ± SEM. *V*_1/2_: voltage of half‐maximally activated or inactivation, *k*: slope factor.

a *P *<* *0.01 vs WT.

### 3.4 Co‐IP study

**Figure 4:**
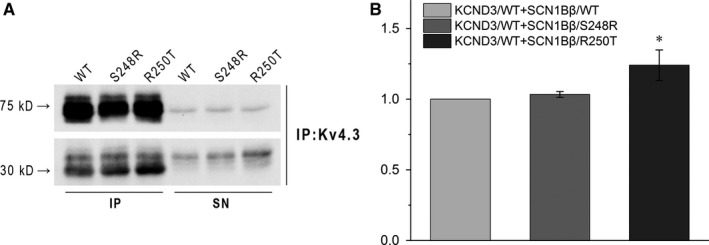
Co‐immunoprecipitation study indicated direct interaction of KCND3 and SCN1Bβ subunits. HEK293 cells were co‐transfected with KCND3/WT and SCN1Bβ (WT, S248R, R250T). Cells were lysed and total protein extracts were immunoprecipitated using anti‐KCND3 and then immunoblot with anti‐KCND3 and anti‐SCN1Bβ. A: Representative western blots of KCND3 (75 kD arrow) and SCN1Bβ (30 kD arrow). IP: immunoprecipitated pellet; SN: supernatant. B: Percentage of SCN1Bβ (WT, S248R, R250T) co‐immunoprecipitation related to the total amount of KCND3/WT immunoprecipitated. *P <* *0.05 vsWT

To test whether SCN1Bβ has some direct effects on Kv4.3, we next used Co‐IP to assess the relationship. KCND3/WT was co‐expressed with SCN1Bβ/WT, SCN1Bβ/S248R or SCN1Bβ/R250T in HEK293 cells and isolated by pull‐down using an antibody to Kv4.3. Figure 4A showed the association between Kv4.3 (~75 kD, top) and SCN1Bβ (~30 kD, bottom) when co‐expressed. Compared with KCND3/WT + SCN1Bβ/WT, co‐expressed with SCN1Bβ/R250T resulted in a significant increase of SCN1Bβ to Kv4.3. However, the amount of SCN1Bβ interact with Kv4.3 was not significant different between WT and S248R (Figure 4B).

## 4 DISCUSSION

In the present study, we characterized two mutations in SCN1Bβ among four families with ERS using patch‐clamp technique and Co‐IP. Electrophysiological study showed that except some minor changes in sodium current, SCN1Bβ/R250T co‐expressed with KCND3 resulted in markedly greater I_to_ current density when compared with SCN1Bβ/WT + KCND3. Electrophysiological properties showed that co‐expressed with SCN1Bβ/S248R or SCN1Bβ/R250T produced I_to_ current with altered kinetics of steady‐state inactivation and recovery from inactivation.

I_to_ plays an important role in the early repolarization phase and abbreviates action potential duration.^19^ Inhibition of I_to_ exerts an ameliorative effect in the setting of ERS by producing an inward shift in the balance of current during the early phases of the epicardial action potential.^32^ Our results showed that S248R and R250T mutations in SCN1Bβ could increase I_to_ channel activities when co‐expressed with KCND3. As both of the mutations of SCN1Bβ we described resulted in an increase of I_to_ channel current, we recognized that these mutations might contribute to ERS. However, whether the degree of augment in I_to_ current is correlate with the clinical presentation and prognosis of ERS is still unknown.

SCN1B gene encodes sodium channel beta1 subunit (Na_v_β1) and sodium channel beta1b subunit (Na_v_β1b), which serves as auxiliary subunits of Na_v_1.5. In 2002, Deschenes et al^33^ found that Na_v_β1 increased the current density of I_to_ by modulating the gating kinetics of Kv4.3. Silencing of Na_v_β1 produced a reduction in Kv4.2, Kv4.3 and KChIP2 mRNA and protein.^34^ These findings suggested a structural and functional association between Na_v_1.5 and Kv4.3 via Na_v_β1. Recently a study reported that R214Q mutation in SCN1Bβ linked to Brugada syndrome and sudden infant death syndrome via a combined loss‐of‐function of Na_v_1.5 current and gain‐of‐function of I_to_ current,^35^ which indicated a similar modulation effect of Na_v_β1b on Kv4.3 as Na_v_β1 does.

In the present study, our results showed that co‐expressed with SCN1Bβ/S248R or SCN1Bβ/R250T increased I_to_ current density by slowing down steady‐state inactivation and accelerating recovery from inactivation compared with co‐expressed with SCN1Bβ/WT as previous study. However, no significant sodium current density was observed in this study thought some minor changes in channel gating kinetics. The probands included in our study showed slurred or notched J‐wave on surfaced electrocardiogram. One of underlying mechanism of J‐wave is prominent voltage gradients in early repolarization between endocardium and epicardium,^36^ which partly due to significant downregulation of I_to_ in endocardium in comparison with that of midmyocardium and epicardium.^37^ Thus, gain‐of‐function mutation of I_to_ channel current in epicardium was expected to produce similar voltage gradients and J‐wave.

The effects of SCN1Bβ variants on Na_v_1.5 currents had been described earlier. Some studied reported that SCN1Bβ variants reduced sodium currents by accelerating recovery from inactivation and decreasing the slow inactivation rate.^29^, ^38^ In contrast, the mutations we reported in the present study could not alter Nav1.5 channel currents significantly. We have noted a previously study identified the same mutations of SCN1Bβ in three asymptomatic members from a family with Brugada syndrome and sick sinus syndrome.^39^ Together, it suggested that the S248R and R250T mutations of SCN1Bβ maybe not pathogenic to Na_v_1.5 function.

Both of the mutations, S248R and R250T, located at the C‐terminal of SCN1Bβ. This hydrophobic region included residues from 243 to 262, which serve as a transmembrane domain.^40^ To date, few mutations in this area were characterized by functional analysis. In present study, we observed an influence in steady‐state inactivation and recovery from inactivation of I_to_ currents. Previously study reported that co‐expressed with SCN1Bβ/WT slowed recovery of I_to_ from inactivation when compared with that of KCND3/WT alone.^35^ Besides, Co‐IP study showed an impaired interaction between KCND3 and SCN1Bβ/R250T. Therefore, we hypothesis that mutation in this area may affect association between KCND3 and SCN1Bβ, either in direct or indirect manners.

In summary, we found that the S248R and R250T mutations in SCN1Bβ gene causes gain‐of‐function of I_to_ by associated with Kv4.3. Our findings suggested that the mutations maybe underlie the ERS phenotype of the probands, and SCN1Bβ maybe one of the possible modulatory genes associated to ERS.

## CONFLICT OF INTEREST

All authors have completed and submitted the ICMJE Form for Disclosure of Potential Conflicts of Interest.
